# The Utilisation of Tannin Extract as a Dietary Additive in Ruminant Nutrition: A Meta-Analysis

**DOI:** 10.3390/ani11113317

**Published:** 2021-11-19

**Authors:** Yulianri Rizki Yanza, Ainissya Fitri, Bambang Suwignyo, Elfahmi, Nanik Hidayatik, Nur Rochmah Kumalasari, Agung Irawan, Anuraga Jayanegara

**Affiliations:** 1 Animal Feed and Nutrition Modelling (AFENUE) Research Group, IPB University, Bogor 16680, West Java, Indonesia; yryanza@edu.uir.ac.id (Y.R.Y.); ainissya25@gmail.com (A.F.); nurrkumala@gmail.com (N.R.K.); a.irawan@staff.uns.ac.id (A.I.); 2 Department of Biology Education, Faculty of Teacher Training and Education, Universitas Islam Riau (UIR), Pekanbaru 28284, Riau, Indonesia; 3 Department of Animal Nutrition, Poznań University of Life Sciences, 60637 Poznan, Wielkopolskie, Poland; 4 Research Center for Biotechnology, Research Organization for Life Sciences (LIPI)—National Research and Innovation Agency (BRIN), Cibinong 16911, West Java, Indonesia; 5 Department of Animal Nutrition and Feed Science, Gadjah Mada University, Sleman 55281, Yogyakarta, Indonesia; bsuwignyo@ugm.ac.id; 6 School of Pharmacy, Institut Teknologi Bandung, Bandung 40116, West Java, Indonesia; elfahmi@gmail.com; 7 Faculty of Veterinary Medicine, Airlangga University, Surabaya 60115, East Java, Indonesia; nanik.h@fkh.unair.ac.id; 8 Department of Nutrition and Feed Technology, IPB University, Bogor 16680, West Java, Indonesia; 9 Vocational School, Universitas Sebelas Maret, Surakarta 57126, Central Java, Indonesia; 10 Department of Animal and Rangeland Sciences, Oregon State University, Corvallis, OR 97331, USA

**Keywords:** tannin extract, ruminant, methane, milk, N utilisation, rumen, meta-analysis

## Abstract

**Simple Summary:**

Tannin has been extensively assessed for its potential and utilisation as a ruminant feed additive in recent years and is becoming important due to its beneficial effects on modulating ruminant performance and health and mitigating methane emissions. However, evidence concerning the effect of tannin in extracted forms on ruminants appears to be inconclusive on whether it can genuinely provide either beneficial or detrimental effects for ruminants. Moreover, the effects of various sources, types of tannin extract, or appropriate levels of supplementation on ruminants remain unclear. Therefore, there is a need for a systematic evaluation concerning the effects of tannin extract on rumen fermentation, digestibility, performance, methane emissions, and metabolism of ruminants.

**Abstract:**

The objective of this meta-analysis was to elucidate whether there are general underlying effects of dietary tannin extract supplementation on rumen fermentation, digestibility, methane production, performance, as well as N utilisation in ruminants. A total of 70 papers comprised of 348 dietary treatments (from both in vivo and in situ studies) were included in the study. The database was then statistically analysed by the mixed model methodology, in which different experiments were considered as random effects and tannin-related factors were treated as fixed effects. The results revealed that an increased level of tannin extract inclusion in the diet lowered ruminant intake, digestibility, and production performance. Furthermore, the evidence also showed that an increased level of tannin extract decreased animal N utilisation where most of rumen by-pass protein was not absorbed well in the small intestine and directly excreted in the faeces. Due to the type of tannin extract, HT is more favourable to maintain nutrient intake, digestibility, and production performance and to mitigate methane production instead of CT, particularly when supplemented at low (<1%) to moderate (~3%) levels.

## 1. Introduction

Tannin is known for its anti-nutritional properties due to its detrimental effects on feed intake, rumen microorganisms, nutrient utilisation, and production performance of ruminant livestock, particularly when present at a high concentration in the diet [1]. However, when present at a low to moderate level, tannin may provide beneficial effects to modulate ruminant performance, health, and environmental sustainability [2]. Its molecular structure enables it to modulate ruminal fermentation by binding to protein through hydrogen bonds and forming a tannin–protein complex, thus influencing protein degradation in the rumen [3]. The tannin–protein bound in the rumen is stable at a normal pH environment and resistant to rumen microbial degradation, but it dissociates at a low pH environment in the abomasum [4]. Thus, tannin supplementation commits to lowering the amount of protein that is degraded in the rumen and increases the flow of by-pass protein to the small intestine. Tannin may also alleviate the toxic effect of high rumen ammonia concentration and improve nitrogen efficiency [5,6].

Another beneficial effect of tannin is its ability to decrease enteric methane emissions [7]. Enteric methane emissions are an important issue to consider since ruminants contribute to approximately 17% of global methane emissions or about 47% of the global livestock sector for global greenhouse gases [8,9]. A number of experiments have demonstrated the methane-mitigating property of tannin. For instance, Zhang et al. [10] found that the supplementation of 60 g/kg extracted hydrolysable tannin (HT) from Chinese nutgall decreased methane production up to 30–36% in sheep, while Pineiro-Vazquez et al. [11] found that the supplementation of 30 g/kg extracted condensed tannin (CT) from Mimosa decreased sheep methane production up to 38%. However, there were contrasting results regarding the methane mitigating effect of tannin; some other experiments did not observe any reduction in the methane emissions of ruminants after being supplemented with tannin. These variations depend on the level, type of tannin applied, plant sources, and form of tannin [7].

Tannin may be supplemented into the diet either as tannin-containing plants or as its extracted form. The use of tannin extract instead of tannin-containing plants is typically preferable for a large-scale and commercialised ruminant production system such as in a feedlot. The commonly used tannin extract originates from acacia, quebracho, chestnut, and mimosa. Such various sources of tannin extract and different doses of dietary supplementation may lead to their inconsistent and highly variable effects on ruminant production such as nutrient intake, digestibility, production performance, methane emissions, product quality, and other parameters. Therefore, there is a need for a systematic evaluation concerning the dietary supplementation of tannin extract in ruminants. The objective of this study was to examine the effects of tannin extract supplementation at various levels and sources (types) on nutrient intake, rumen fermentation, digestibility, methane production, blood metabolites, production performance, and nitrogen utilisation of ruminants by employing a meta-analysis method.

## 2. Materials and Methods

### 2.1. Database Development

**Figure 1:**
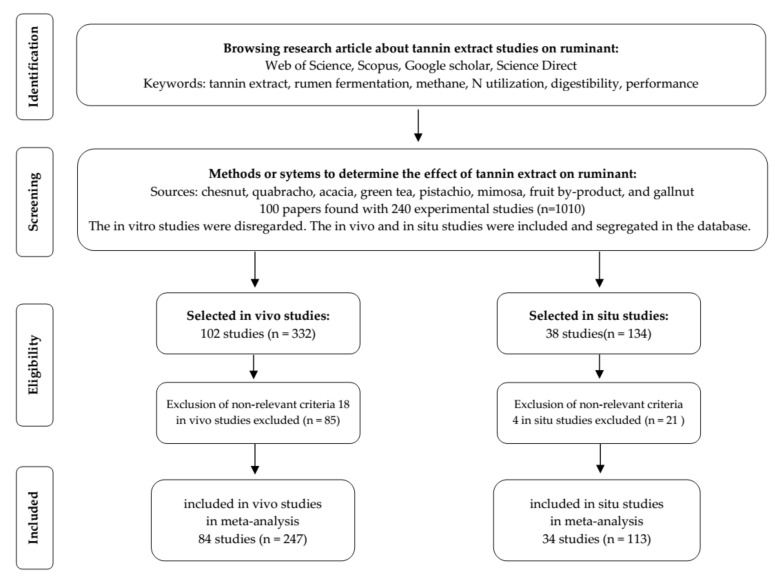
Diagram flow for selection of the studies on the influence of tannin extract on ruminants.

A database was constructed from various experiments reported in the literature where tannin extract was supplemented into ruminant diets. All constructed data were based on in vivo and in situ experiments (did not include in vitro experiments), obtained from various electronic journal platforms such as Web of Science, Scopus, Google Scholar, and Science Direct. The selection of studies included in the database is graphically presented in Figure 1.

A total of 118 experimental studies, both in vivo and in situ, from 70 papers and comprised of 360 dietary treatments were finally integrated into the database (summarised in Table 1). Experimental studies were treated individually even when published within an article. The database was segregated into two categories based on the study methods, i.e., in vivo studies (84 experiments, 247 treatments) and in situ studies (34 experiments, 113 treatments). Animals that were involved in the in vivo and in situ experiments were large ruminants (lactating dairy cows, heifers, and beef cattle, both steers and bulls) and small ruminants (goats and sheep). Parameters included in the meta-analysis were nutrient intakes such as the digestibility of dry matter (DMD), organic matter (OMD), crude protein (CPD), and neutral detergent fibre (NDFD); production performance such as weight gain and feed efficiency; methane production; milk production and composition; rumen fermentation and microbial profiles; ruminal feed disappearance; blood plasma metabolites; N utilisation; and urinary purine profile.

The tannin form was specified as HT, CT, or unspecified or represented a mixture of HT and CT. The unspecified tannin then was categorised as CT or HT based on the primary tannin content. Overall, the sources of extracted tannin were obtained from chestnut, quebracho, acacia, green tea, pistachio, mimosa, fruit by-product such as grape pomace and pomegranate peel, gallnut, as well as commercial or unspecified tannin. Extracted tannin sources from acacia, *Cistus ladanifer* L., grape pomace, mimosa, pomegranate peel, quebracho, and *Vaccinium vitis idaea* were classified as a source of CT. Meanwhile, extracted tannin sources from chestnut, gallnut, green tea, pistachio, valonia, and tara were classified as a source HT. The supplementation level of tannin extract was presented as g/kg DM of feed, and measurements expressed in other units (mg/mL, % *v*/*v*, or % *w*/*v*) were converted to g/kg DM from available information in the papers. Supplemented tannin extract in the diet ranged from 0 (typically in the control diet) to 140 g/kg DM. The data points of animals treated with polyethylene glycol were not included in the database since this compound is known to be a tannin-deactivating agent [12].

The measurement of CH_4_ emissions in the in vivo experiments was performed by using a respiration calorimetry system equipped with an infrared CH_4_ detector. The units for milk composition and milk N utilisation were converted and presented as g/100 g, while the units for rumen fermentation profiles, rumen ammonia, milk urea N, or blood plasma were converted and presented as mmol or mg/dL. The unit for production performance, digestibility, and milk production parameters was presented as g/d, kg/d, or converted to g/kg metabolic body weight (g/kg BW^0.75^). The unit for the in situ degradation kinetics was uniformed in percentage (%) unit. The statistical summary of the database is presented in Table 2.

### 2.2. Statistical Analysis

The database was analysed by employing the mixed model methodology [79,80], using the MIXED procedure of SAS software (version 9.2, SAS Institute Inc., 2008). Different experiments were considered as random effects and tannin-related factors (either concentration or type of tannin) were treated as fixed effects, followed Jayanegara et al. [12] and Yanza et al. [9] with some modifications. The assessment of the tannin extract supplementation level and tannin type (CT or HT) was accomplished with the following statistical model:

*Y_ij_* = *µ + s_i_* + *τ_j_* + *sτ_ij_* + *B_0_* + *B_1_X_ij_* + *B_2_X^2^_ij_* + *b_i_X_ij_* + *e_ij_*

where *Y_ij_* = dependent variable, *µ* = overall mean, *s_i_* = random effect of the -*i*th experiment, *τ*_j_ = fixed effect of the -*j*th level of factor *τ*, *sτ_ij_* = random interaction between the -*i*th experiment and the -*j*th level of factor *τ*, *B_0_* = overall intercept across all experiments (fixed effect), *B_1_* = linear regression coefficient of Y on X (fixed effect), *X_ij_* = value of the continuous predictor variable (tannin extract level), *b_i_* = random effect of study on the regression coefficient of *Y* on *X* in study -*i*, and e*_ij_* = the unexplained residual error. The CLASS statement was declared based on the tannin type and the study variable since they did not contain any quantitative information. The RANDOM statement was declared based on different studies included. The number of replicates in the studies was declared in the WEIGHT statement available in SAS as performed by Jayanegara et al. [12] and Yanza et al. [9]. The model was considered significant at *p* ≤ 0.05 or tends when the *p*-value was >0.05 and ≤0.10.

## 3. Results

The addition of tannin extract did not affect ruminant performance, such as average daily gain expressed as gram/d (ADG), gross energy intake (GEI/BW^0.75^), digestible energy intake (DEI/BW^0.75^), and metabolizable energy intake (MEI/BW^0.75^) (Table 3). However, when expressed as ADG/DMI (g/kg DM intake; feed efficiency), animal weight gain tended to increase with the increased tannin extract concentration following a quadratic response (*p* = 0.092). Concerning nutrient intake, although the OMI and CPI were not affected by tannin extract supplementation, daily DMI (kg/d) and DMI per kg metabolic body weight (DMI/BW^0.75^) were decreased by quadratic response (*p* = 0.002) and linear response (*p* < 0.001), respectively. The concentration of tannin extract also decreased the daily NDF intake (*p* = 0.025) as well as CPI/BW^0.75^ (*p* = 0.005) and NDFI/BW^0.75^ (*p =* 0.003) in a linear response. The OMI/BW^0.75^ (*p =* 0.058) tended to decrease linearly by the increased level of tannin extract supplementation. The DMD, OMD, CPD, and NDFD digestibility were also decreased with increased levels of tannin extract by quadratic responses (*p* < 0.010). In regard to the type of tannin supplementation (CT vs. HT), there were significant interaction on the NDFD (*p* = 0.044) and a tendency (*p* = 0.096) of interaction on NDFI/BW^0.75^.

Methane emissions expressed as CH_4_/DMI and CH_4_/BW^0.75^ were lowered by the increased level of supplementary tannin extract with a linear response (*p* < 0.010). Significant responses were also shown on the methane production expressed as CH_4_ (L/d; *p* = 0.047) and CH_4_/BW^0.75^ (L/kg; *p* = 0.046), as well as tended to different for CH_4_/DMI (L/kg; *p* = 0.051) in the case of tannin type. Milk yields expressed in kg/d tended to decrease with increased concentrations of tannin extract (*p* = 0.083) with a quadratic response, but were not affected when expressed as Milk yield/BW^0.75^ and Milk/DM intake. However, FPCM, solid non-fat, total solid, and urea-N in milk were decreased by the level of tannin extract supplementation (*p* ≤ 0.01), where FPCM showed a quadratic response while others showed linear responses. Although there is no effect by tannin extract concentration, protein (*p* = 0.094; tended to be significant) and lactose (*p* = 0.022, significant) content in milk were influenced by the different types of tannin extract.

The rumen fermentation parameters such as pH and Iso-C5 proportion were not affected by tannin extract supplementation (Table 4). However, the TVFA, C2, C5, and the ratio of C2:C3 were decreased by increasing the concentration of tannin extract (*p* < 0.01), where the NH3, TVFA, C2, and the ratio of C2:C3 showed a linear response and C5 had a quadratic response. In contrast, C3, Iso-C4, and C4 proportions were increased by the concentration of tannin extract supplementation (*p* < 0.050), where Iso-C4 showed a quadratic response while C3 and C4 showed a linear response for their models. Concerning the rumen microbial population, the levels of tannin extract supplementation had no significant effect on the bacterial population but tended to linearly decrease the protozoa population (*p* = 0.058). Nonetheless, only C2 and C4 had significant differences by the type of tannin extract (*p* < 0.050). Meanwhile, digestibility aspects such as ruminal total N, ruminal OM-N, ruminal total protein, and intestinal protein were decreased following a linear response due to increasing the concentration of tannin extract (*p* < 0.05), but no effect was observed on duodenal total protein digestibility.

The plasma urea-N (PUN) was decreased by a quadratic response (*p* = 0.002) (Table 5) when the supplementation of tannin extract increased and tended to be significantly influenced by the type of tannin extract (*p* = 0.089). Although the albumin was not influenced by the tannin extract concentration, the type of tannin tended to affect the albumin concentration in the blood plasma (*p* = 0.060). Concerning N utilisation, the concentration of tannin extract did not affect milk-N and urine-N output. However, the faeces-N output was significantly increased linearly by the level of tannin extract supplementation (*p* < 0.001). N retention was also increased by the concentration of tannin extract with a quadratic model (*p* < 0.001) and was significantly influenced by different types of tannin extract supplementation (*p* = 0.012). However, the ENU tended to decrease by the concentration of tannin extract with a quadratic response (*p* = 0.070). Based on urinary purine, the concentration of allantoin and microbial N supply were not influenced by the level of tannin extract supplementation in ruminants. However, uric acids and purine derivative concentration tended to be lowered by the level of tannin extract supplementation (*p* < 0.010) and significantly depended on the type of tannin extract (*p* < 0.001). Meanwhile, the effectiveness of microbial protein supply (EMPS) was significantly lowered by the increase in concentration of tannin extract (*p* = 0.043), and the type of tannin significantly affected the EMPS reduction (*p* < 0.001).

In the in situ studies, the concentration of tannin extract supplementation significantly decreased *a*, *a* + *b*, and *c* coefficients followed by a decrease in the ERD percentage at 2%, 5%, and 8% (*p* < 0.001) of DM and CP (Table 6). The coefficient of the non-soluble fraction (*b* coefficient) of DM and CP was increased quadratically by the level of supplemented tannin extract (*p* ≤ 0.001), which was also influenced by the type of tannin (*p* = 0.072 and *p* < 0.001, respectively). On the other hand, *a*-dm, *a*-cp, ERM 2%, and ERM 8% of DM were significantly affected by the type of tannin (*p* < 0.050). The *a* and *a* + *b* of CP were also assigned for the type of tannin (*p* < 0.005). Meanwhile, there was no significant dependence on ERM percentages of CP degradability. Moreover, the concentration of tannin extract decreased the ID but increased the RUP percentage, and both variables were changed in a linear response (*p* < 0.001).

## 4. Discussion

### 4.1. Influence of Tannin Extract on Performance, Digestibility, Rumen Parameters, Milk Production, and Methane Production

Investigations on the influence of dietary tannin extract supplementation in animals have been growing massively in the last two decades, especially on ruminants [12]. The intervention with tannin obtained large variability in the outputs, whether beneficial and/or detrimental on ruminants’ health and production. Tannin is generally known for its capability to bind with protein in feed, forming a tannin–protein complex that is stable at ruminal pH conditions but dissociates at abomasal acidic pH or duodenal alkaline pH. Accordingly, most of the tannin–protein complex is skipped from ruminal protein degradation and is non-denatured protein for further metabolic processes in the intestine, which is beneficial for metabolism efficiency, optimising dietary energy utilisation when supplemented at appropriate doses [4,81,82]. Another beneficial effect is the toxic effect of tannin that could diminish undesirable ruminal microorganisms involved in methane formation, resulting in lower methane production [7]. Nonetheless, due to the presence of other bioactive molecules in the whole plant that might interfere with the tannin effect such as phenolic acid, flavonoids, diterpenes [83,84], saponins [85], lipids [9], and essential oils [86], studies regarding the effect of tannin on ruminants have been moving forward to specifically determine the influence of tannin in extracted or purified form on ruminant methane production, digestibility, and performance [87,88]. It is expected that the effects of extracted tannin on those parameters would be more obvious corresponding to the type of tannin used, i.e., CT and HT.

In this meta-analysis, supplementation with tannin extract (HT and CT) had an adverse effect on the nutrient intake of ruminants. It is generally known that tannin in the diet influences ruminant palatability. Thus, under this aversion, a decrease in feed intake and rate of digestion in the rumen might occur [89]. On the contrary, some studies reported a non-detrimental effect of tannin extract on ruminant intake [16,26,51]. Meanwhile, decreases in nutrient intake were more obvious in the present study, probably because ruminants had a limited adaptation period to the supplementary tannin extract in the diets. Similar results were reported in our previous meta-analysis study where tannin supplementation impaired ruminant dry matter intake and performance [7]. We suspected that the unaffected nutrient intake may be attributed to the presence of tannin extract in a low concentration, about 0.5–3% of the total diet [11]. Another reason that should be noticed is that some treated animals were fed a diet composed of molasses, which can improve animal palatability [25]. Thus, the effect of tannin on animal palatability was resolved. However, the decrease in nutrient intake was concomitant with a depression in nutrient digestibility (Table 4), especially on NDF. Tannin extract tended to impair the NDF intake and digestibility rate, in which the type of tannin (CT and HT) might also influence the ruminant digestibility rate differently. This is plausible because tannins are acknowledged for their detrimental effects on ruminant digestibility by coating the physical attributes of feed particles due to the tannin–fibre or tannin–protein complex binding. In addition, tannin also caused intoxication in ruminal microorganisms, especially fibre-degrading bacteria, thus preventing them from rumen degradation [51,90,91]. In accordance with the type of tannin, we assume that the condensed tannin exerted a greater repercussion on a nutritional and digestibility perspective than HT. This is because CT had a greater affinity for more solid feed particles and is more difficult to hydrolyse than HT, which is easier to degrade by rumen microbes.

Such conditions also influenced methane production, which was confirmed by the reduced methane production in the present study, and this was associated with the decrease in ruminal fibre degradation. Limited fibre degradation as a result of fibre–tannin bonding is unfavourable to synthesising optimum VFA by rumen microorganisms; hence, the H_2_ supply is also limited for methanogens to perform methanogenesis. Therefore, the increasing level of tannin extract in the diet tremendously suppressed rumen methane formation due to the decrease in acetate formation from pyruvate [7,92], although there was no significant effect on total VFA by increasing the supplementary level of tannin extract. The enhancement of propionate concentration occurred by the lack of activity of acetogenic bacteria due to tannin biological activity, while H_2_ utilisation was shifted to propionate formation where free-H_2_ is more approbatory for propionic bacteria agents [83,93]. Hence, a lowered C_2_:C_3_ ratio was also confirmed in the present study.

If we compare the effectiveness between tannin types on reducing methane production, HT seems to have a greater ability to reduce methane production than CT. According to Jayanegara et al. [81], a decrease in methane production is strongly related to the protein precipitation degree caused by tannin–protein complexes. In such a way, HT is more susceptible to microbial degradation involved in the methanogenesis process (fibrolytic bacteria and methanogens) due to the fact that the HT hydrogen bond is easily attached to microbial cells or enzymes that are toxic to rumen microbes; thus, this condition may impair the microbial metabolism. Although the bacterial population in the present study was not clearly affected by tannin biological activity, tannin is generally known to decrease bacterial attachment to plant particles and cause subsequent decreases in N and NDF digestibility [45,81,94]. Perhaps this condition could explain the unaffected VFA concentration in the rumen by the increased level of tannin in the diet. Moreover, such tannin mechanisms could be associated with the decrease in the protozoa population where this microbe is involved in methanogenesis [31,95,96].

The decrease in ammonia (NH_3_) concentration also showed an obvious relationship with the increased level of tannin, whereas the feed particles that formed fibre–tannin and protein–tannin complex bonds are difficult to degrade by proteolytic bacteria. Thus, protein and amino acids protected by tannin to pass rumen fermentation are favourable because this would increase protein absorption in the small intestine, which in turn increases N use efficiency. On the contrary, most reports showed that most of the rumen by-pass protein and amino acids were undigested in the small intestine due to the strong protein–tannin molecule bonds that are difficult to break down by the intestinal enzyme. This explains why somehow N and amino acid supplies for animal metabolism were lower than the expectation. Likewise, although tannin is propitious in decreasing methane production, both tannin types may be supplemented in a low dose; hence, their adverse effect on performance and nutrient digestibility can be averted [51].

Moreover, our meta-analysis has shown that ruminant performance was also decreased. The decrease in animal weight gain (ADG) was robustly correlated to the decreased nutrient intake and digestibility, but feed efficiency (ADG/DMI) tended to be increased. The lower ADG might reflect the negative association between tannin intervention and nutrient intake and digestibility that might not meet the animal growth requirements [34]. On the other hand, the decrease in milk yield in the present study was not observed as of kg/DM intake or milk yield/metabolic BW (g/kg^0.75^). Although there was a potential decrease in milk production (kg/d), which might not be related to tannin intervention directly, this aspect needs further assessment. The fat protein corrected milk (FPCM), which represents the general model of milk fat and protein composition as well as general milk yield (kg/d), was consistently lowered due to increasing levels of tannin extract supplementation. Toral et al. [73] reported that the inconsistent effect of tannin on milk production is probably related to ruminant species, dietary treatment period, type of tannin, and dose. However, no significant difference was noticed in the present study regarding milk yield. It was in the range of FCPM value according to the Dutch feeding system for dairy cows as reported by Herremans et al. [42], which is between 23.9 and 26.1 FPCM. Although the total solid and solid non-fat were also influenced by the level of tannin supplementation, they were decreased only if the dairy animal was fed with a high dosage of tannin extract. This finding emphasised that tannin inclusion in the diet would only slightly affect the milk yield and total solid in milk with or without fat composition, where tannin did not increase the quantity of digestible proteins, thus explaining milk N stability [42,56].

Milk yield and milk composition results were inconsistent, but the changes in milk components such as protein and lactose were largely dependent on the different types of tannins. HT and CT showed different effects on protein and lactose contents in milk, whereas HT seems to have a better beneficial value compared with CT. This is likely because the hydrogen bond of HT derivative in the rumen is weaker, thus it is easier to degrade, with the consequence that the by-pass protein is preserved for further metabolism processes, e.g., glucose and protein deposition in milk is higher when compared to the case of CT. Such HT inclusion in ruminant diet may provide better protein and lactose composition in milk rather than CT [73]. Above all, it is critical to consider the type and levels of tannins supplemented by dairy cows.

### 4.2. Influence of Tannin Extract on Ruminal N Digestibility, Blood Plasma, N Utilisation, and Urinary Purine Derivative of Ruminants

Since the beneficial effects of tannin are primarily known to protect the feed by-pass protein (degradable) and distribute their amino derivative to further metabolism processes, the protected protein was expected to be absorbed in the small intestine and accumulated in the liver. Plasma urea nitrogen (PUN) and albumin concentration in the blood are considered as parameters to clarify animal protein status [18]. The albumin concentration from CT and HT interventions might appear differently due to the difference in digestibility index associated with them. Meanwhile, the decrease in PUN concentration occurred due to the undissociated by-pass protein in the small intestine. Moreover, PUN is not absorbed but produced in the liver or from ammonia coming from the rumen or gut epithelium or amino acids used in the liver for gluconeogenesis. For example, Orlandi et al. [59], who observed steers and offered *Acacia mearnsii* tannin extract at the rates of 20, 40, or 60 g/kg DM, found a linear decrease in ruminal ammonia while the faecal nitrogen (N) excretion, N retention, and the efficiency of N utilisation increased. In their report, they found an increase in N duodenal flux, α-amino N, and non-ammonia non-microbial N. However, Wischer et al. [76] also found an increase in faecal-N but without any difference in N retention and urinary N in sheep treated with chestnut and valonea tannin at 20 g/kg DM.

Although the increased level of tannin showed a positive relationship with N retention and the efficiency of N utilisation in ruminants, nevertheless, instead of being absorbed, most of the protein–tannin complexes were not dissociated in the small intestine, which is also confirmed in the present study. Consequently, N excretion might also increase, thus expected higher growth did not occur. When animals are fed with high dietary protein in parallel with elevated tannin supplementation, unfortunately, the intestinal enzymes are disabled to degrade most of those tannin–protein complexes, making it less available for further metabolism. Both tannin types had similar effects on the decrease in PUN. The present findings agreed with Henke et al. [1], who observed the effect of quebracho tannin extract at 15 and 30 g/kg DM on dairy cows. They suggested that tannins are less effective at improving feed intake and protein use efficiency. However, if the tannin–protein complexes disassociated post-ruminally and amino acids could be absorbed in excess, absorbed PUN would be expected to be similar in cattle fed an excessive protein diet without tannin [47].

Sequential effects by increasing the level of tannin extract presence in the diet cannot be evaded. It can be seen by the indirect effect on the reduction in milk urea N (MUN). The MUN concentration is a necessary parameter to estimate and monitor the nutritional status of lactating dairy cows as well as to improve dairy herd nutrition [36]. This condition is believed to be correlated with the effect of tannin inclusion that influences lower N intake, provides insufficient absorbable N in the small intestine, and is distributed below the required concentration in the blood; hence, the MUN deposition in milk was also reduced. Although N retention was potentially increased, most of the protein was poorly absorbed due to tannin extract supplementation, indicated by the increase in faecal N concentration. Although N-urine was not affected, the uric acids, purine derivatives (PD), and effectiveness of microbial protein supply (EMPS) were decreased. Urinary PD is commonly used as an indicator for the effectiveness of rumen MCP synthesis [36]. A lower urinary PD excretion pinpoints that the tannin extract reduces the microbial protein reaching the duodenum. In such a case, it showed that by-pass protein was not thoroughly absorbed and distributed for metabolic purposes as it was shown to increase N in faeces and urine as well as the concentration of uric acids, PD, and EMPS rates. Koenig et al. [36] suggested that amino acids from feed absorbed in excess or with an imbalanced profile with maintenance production requirements are extracted and deaminated in the liver and the N is also excreted in the form of urea N in urine. Due to the different biological characters of tannin, it seems that the CT tannin–protein bond is difficult to hydrolyse post-ruminally; therefore, feed protein bonded with the HT tannin was more available to be absorbed in the ruminant hindgut.

### 4.3. Influence of Tannin Extract on Kinetics Degradability In Situ 

The distinct effects of tannin extract on ruminant digestibility can be observed thoroughly from the kinetics degradability of in situ experiments. The decrease in non-soluble fractions of DM and CP indicates an inhibitory effect on endoglucanases and cellulose degradation of feed particles due to the protein–tannin or fibre–tannin complex bonds. Moreover, some proteolytic bacteria are noticed to be able to modify their metabolism, i.e., adapt with a selective advantage environment to grow in the presence of phenolic compounds such as tannin [97,98]. Thus, rumen degradation was potentially reduced by the increased levels of tannin because ruminal microbes are also sensitive to the presence of tannin extract. Our evidence showed that a low dosage of tannin extract inclusion might not adversely affect the rumen bacterial population. However, they persistently impair ruminant digestibility and productivity. The presence of tannin extract is toxic to several species of rumen bacteria. Therefore, inhibitory effects on protein proteolysis often occur, and in some conditions, the polymer–tannin bond fails to be absorbed as rumen undegradable protein (RUP) in the intestine. Nasehi et al. [57] reported that tannin reduced the ruminal degradability of plant proteins and enhanced the intestinal bioavailability of amino acids in ruminants. By contrast, our evidence showed that the presence of tannin extract negatively influenced rumen protein degradability and total tract apparent digestibility. Concerning the difference in effectivity between tannin types, the reduced ruminal degradability was also influenced by the difference in the biological activity of tannin as we described above (Section 4.1).

### 4.4. Noticeable Effect by the Divergence between Tannin Extracts

In the present study, types of tannin were distinguished into CT and HT as those types have different chemical properties [12]. HT is a hydroxyl group of which they are partially, or fully, esterified with either gallic or hexahydroxydiphenic acid and may have long chains of gallic acid coming from the central glucose core [99]. HT is hydrolysed into their constituent phenolic acids with acid or enzymes. Meanwhile, CT includes polymers formed by the condensation of flavans molecules such as procyanidin, or higher oligomers of substituted flavan-3-ols, but they do not contain any sugar residues [100]. CT monomers are favourable to link with carbon bonds and difficult to break down where the molecule bond stability is vigorous. The molecules can be broken down by heating or strong acids.

However, their mechanism can be explained chemically based on the data analysed in the present study. HT had a stronger protein precipitation ability than CT; thus, methane emissions were decreased effectively, and by-pass protein might escape from the rumen. However, a higher level of HT presence in the diet may not effectively alter ruminant metabolism in a further condition since the HT–protein or HT–fibre bonds are hydrolysed by ruminal microbes or intestinal enzymes. HT might camouflage the bonds of protein or fibre; hence, in such a way the absorbable nutrient might escape for further metabolism processes. Meanwhile, when CT bonded with carbonic groups of feeds, the ruminal microbes found it difficult to break down the CT–protein or CT–fibre in the rumen due to their solid bonds. Escaped rumen CT–feed bonds were also difficult to degrade. Therefore, the adverse effect of CT on ruminant digestibility is potentially greater than HT. Moreover, although the faecal-N and urinary-N were increased, HT seems to support N retention more than CT due to their sequential effects before escaping the rumen. N supply and available amino acids might be greater when ruminants are fed a diet with HT supplementation compared to CT. This condition might also reflect on animal production such as milk yield and milk composition. However, it should be underlined that the presence of HT can be absorbed in the digestive tract to some extent, whereas HT consumption with excessive amounts can be toxic to ruminants [12]. On the other hand, CT is notably vigorous for ruminal microbial or digestive tract enzymes to absorb. Accordingly, the readily absorbable nutrients are limited in the lower gut [41]. Despite their detrimental effects, both types of tannins may provide some beneficial effects if consumed at a low or moderate dosage.

## 5. Conclusions

The present meta-analysis study evaluated experimental evidence concerning the effects of tannin extract in a beneficial perspective on methane emission reduction and providing higher rumen by-pass protein with the appropriate level of tannin extract. However, some detrimental effects such as decreased animal intake, digestibility, and performance also occurred with excessive levels of tannin extract supplementation. Such a condition occurred due to tannin’s ability to limit proteolysis in the rumen and digestive tract; however, the by-pass protein was less available for absorption in the intestine due to strong CT–protein or CT–fibre bonds that were difficult to dissociate. Thus, ruminant weight gain and milk yield were distinctly impaired by tannin. Otherwise, tannin mechanisms on those parameters were also specified by different types of tannin and their chemical properties. HT seems to be more favourable for ruminants instead of CT. However, HT and CT tannin supplementation were distinctly effective at a low dosage of supplementation to enhance more beneficial outcomes.

## Figures and Tables

**Table 1:** Studies included in the meta-analysis of the influence of dietary tannin extract concentration on ruminants.

Nr.	References Nr.	Experiment	Animal	Species and Status	Tannin Source	Tannin Type	Tannin Level (g/kg DM)	Adaptation/Exp.day (d)	Tannin Applied	Basal Feed
1	[2]	in vivo	sheep	Merino	Silvafeed and *A. mearnsii*	CT	0–50	14/26	mixed in diet (TMR)	eragrostis and lucerne hay, and concentrate
2	[3]	in vivo	steer	Jersey	*A. mearnsii*	CT	0–20	14/20	mixed in diet	Tifton hay, corn, soybean meal
3	[6]	in vivo	lamb	South African Mutton × Merino	*A. mearnsii*	CT	0–42	21/60	mixed in diet	eragrostis, lucerne hay, sunflower meal, ground maize
4	[10]	in vivo	sheep	Han × Dorper, small tailed castrated	Gallnut	HT	0–60	14/24	added in diet	corn, soybean meal, wheat bran, rapeseed meal, rice bran, cottonseed meal, DDGS, alfalfa hay, and Chinese wildrye grass
5	[11]	in vivo	heifer	Crossbred	Quebracho	CT	0–40	14/23	added in diet	*Pennisetum purpureum* grass
6	[13]	in vivo	dairy cow	FH	Chestnut and Quebracho	CT and HT	0–18	14/21	mixed with TMR	alfalfa silage, corn silage, cottonseed, rice hulls (replaced with tannin)
7	[14]	in vivo	heifer	FH	Quebracho	CT	0–60	42,248	infusion intraruminally	hay and concentrate
8	[15]	in situ	sheep	Ghezel	Grape pomace	CT	0–60	10/ns	mixed with feed	lucerne hay, wheat bran, and barley grain
9	[16]	in vivo	goat	Boer	Quebracho	CT	0–40	21/27	mixed with feed	grass hay, concentrate
10	[17]	in vivo	dairy cow	FH, lactating, multiparous	*A. mearnsii*	CT	0–16	44,256	administered via rumen-fistula	alfalfa hay, concentrate
11	[18]	in vivo	steer	Weaned Crossbred	Chestnut	HT	0–15	ns/114	supplemented in diet	alfalfa and barley silage
12	[19]	in vivo	dairy goat	Liuyang black nannies, lactating, multiparous	Gallnut	HT	0–9	14/42	mixed in diet (TMR)	forage and concentrate (TMR)
13	[20]	in vivo	steer	FH	*A. mearnsii*	CT	0–50	42,278	mixed in diet	maize silage, soybean meal, canola meal (TMR)
14	[21]	in vivo	heifer	Jersey	Quebracho	CT	0–6	14/47	supplemented in diet	barley grain, barley silage, and canola meal
15	[22]	in vivo	heifer	Angus	Quebracho	CT	0–20	ns/28	supplemented in diet	barley silage, barley grain, soybean meal, and corn gluten meal
16	[23]	in vivo and in situ	dairy cow	FH, lactating	Quebracho	CT	0–4.5	15/28	supplemented in diet	grass silage, corn, beet pulp, corn gluten meal, and wheat bran
17	[24]	in vivo	ewe	Comisana, multiparous	Chestnut and Quebracho	CT and HT	0–52.8	15/28	mixed in diet	barley, corn, wheat bran, soybean mela, beet pulp, and soybean oil
18	[25]	in vivo	ewe	Sarda, multiparous	Chestnut	HT	0–80	21/49	mixed in diet	ryegrass, oat, and white clover
19	[26]	in vivo	dairy cow	FH, lactating	Quebracho	CT	0–30	13/21	added to basal diet	grass silage, maize silage, rapeseed expeller, wheat grain, and concentrate
20	[27]	in vivo	dairy cow	Polish FH	*Vaccinium vitis idaea*	CT	0–140	21/24	supplemented in diet	corn silage, lucerne silage, meadow hay, wheat grain, corn grain, and rapeseed meal
21	[28]	in situ	dairy cow	FH	Chestnut	HT	0–46	21/28	added in diet	lucerne silage, maize silage, grass hay, maize meal, soybean meal, and barley meal
22	[29]	in vivo	ewe	TexelxLacaune crossbreed	*A. mearnsii*	CT	0–20	14/19	added to basal diet	corn silage, pre-dried alfalfa, and soybean meal
23	[30]	in vivo	sheep	-	Cheestnut and Mimosa	CT and HT	0–76.1	15/21	added to diet and mixed with silage	ryegrass
24	[31]	in vivo	dairy cow	Brown-Swiss	*A. mearnsii*	CT	0–14.7	19/23	in pellet form (acacia pellet)	corn silage, grass silage, grass hay, and concentrate
25	[32]	in situ	ram sheep	-	*Cistus ladanifer* L.	CT	0–117	ns	added (mixed) with soybean meal	wheat, barley, maize gluten feed, sunflower meal, and soybean meal
26	[33]	in vivo and in situ	ram sheep	Merino	*Cistus ladanifer* L.	CT	0–30	14/29	added (mixed) with soybean meal	oat straw, manioc, and soybean meal
27	[34]	in vivo	lamb	Merino Branco	*Cistus ladanifer* L.	CT	0–30	15,523	added (mixed) with soybean meal	grass hay, maize, citrus pulp, and soybean meal
28	[35]	in vivo	heifer	Jersey × German Black Pied Lowland	Quebracho	CT	0–60	43,709	infusion intraruminally	grass hay and concentrate
29	[36]	in vivo	dairy cow	FH	Quebracho	CT	0–30	14/21	supplemented in diet	alfalfa hay, corn silage, barley, beet pulp, corn, canola meal, and wheat
30	[37]	in vivo	dairy cow	FH	Quebracho	CT	0–18	33,055	added in diet	alfalfa silage, corn silage, rolled HMSC, corn grain, canola meal, ESMB, soybean meal, cottonseed, soy hulls, and rice hulls
31	[38]	in vivo	sheep	-	Quebracho	CT	0–36.5	22,190	intraruminal infusion and treated soybean meal	alfalfa and grass hay
32	[39]	in vivo	dairy cow	FH	*A. mearnsii*	CT	0–19	2/8 and 14/49	mixed with water, grazing, and stall	ryegrass
33	[40]	in vivo	dairy cow	FH	*A. mearnsii*	CT	0–29	46,813	oral drench and mixed in barley pellet	ryegrass (pasture), barley, and molasses
34	[41]	in vivo	dairy cow	FH	Quebracho	CT	0–30	13/21	mixed in diet	grass silage, maize silage, wheat, rapeseed, and concentrate
35	[42]	in vivo	dairy cow	FH	Oak	HT	0–26	14/21	mixed in grass silage	grass silage, corn silage, beet pulp, rapeseed, and wheat
36	[43]	in situ	ewe	Merino	Tannic acid	HT	0–200	10/ns	treated with soybean meal	grass hay and soybean meal
37	[44]	in situ	ewe	Merino	Quebracho	CT	0–70	ns/51	infusion intraruminally	lucerne hay
38	[45]	in vivo	bull	FH	Pistachio	HT	0–15	14/98	treated with soybean meal	alfalfa hay, corn silage, corn, barley, wheat, soybean meal, and rice bran
39	[46]	in vivo	cattle	FH	*A. mearnsii*	CT	0–6	14/21	mixed in diet	corn silage, corn grain, and soybean meal
40	[47]	in vivo and in situ	heifer	Crossbred, beef heifer	*A. mearnsii*	CT	0–25	21/35	mixed in diet (substituted barley grain)	barley silage, barley grain, and corn DDGS
41	[48]	in vivo	sheep	LeicesterxMerinoxDorset crossbreed	Quebracho	CT	0–60	27/34	oral drench	lucerne hay
42	[49]	in vivo	sheep	PolwarthxTexel wethers crossbreed	*A. mearnsii*	CT	0–60	42,278	infusion intraruminally	ryegrass
43	[50]	in vivo	steer	-	Mimosa and Chestnut	CT and HT	0–15	30/42	supplemented in diet	corn, hay–sorghum, cottonseed hulls, cottonseed meal, and molasses
44	[51]	in vivo	sheep	Santa Inês crossbred	Tannin	CT	0–30	42,278	supplemented in diet	elephant grass, corn, and soybean meal
45	[52]	in vivo	dairy cow	Chinese FH, transition	Chestnut	HT	0–10	ns/42	supplemented in diet	corn silage, alfalfa silage, wheat straw, soybean meal, and corn DDGS
46	[53]	in situ	ewe	Segurena, nonlactating	Tannic acid	HT	0–50	ns	treated with soybean meal	oat hay and barley grain
47	[54]	in vivo	steer	Non-castrated	Quebracho	CT	0–40	14/21	treated with soybean meal	sugar cane bagasse, corn grain, soybean meal, urea, and cottonseed
48	[55]	in vivo	bull	Nellore intact	Tannin	CT	0–75	14/28	treated with soybean meal	sugar cane bagasse, corn grain, soybean meal, urea, and cottonseed
49	[56]	in vivo	dairy goat	-	Pistachio	HT	0–10	14/21	mixed with silage (alfalfa)	alfalfa silage, barley grain, cottonseed meal, and wheat bran
50	[57]	in situ	steer	Talyshi	Green tea	HT and CT	0–19	ns	treated with barley grain	alfalfa hay, wheat straw, and concentrate
51	[58]	in vivo	steer	-	Quebracho	CT	0–45	44,166	added in diet	cottonseed hulls, corn, alfalfa pellet, bermuda-grass hay, and molasses
52	[59]	in vivo	steer	FH	*A. mearnsii*	CT	0–27	42,675	added in diet	oat and concentrate
53	[60]	in vivo	sheep	Santa Ines, male	*A. mearnsii*	CT	0–10	14/21	added in diet	Tifton hay and concentrate
54	[61]	in vivo	sheep	Texel, male	*A. mearnsii*	CT	0–20	14/21	added in diet	oat–ryegrass hay, soybean meal, cracked corn, and wheat bran
55	[62]	in vivo	dairy cow	FH	*A. mearnsii*	CT	0–100	15/21	added in diet	corn silage, corn grain, and soybean meal
56	[63]	in vivo and in situ	sheep	Texel	Chestnut	HT	0–30	14/42	supplemented in diet	orchard grass hay and concentrate based on pea seed
57	[64]	in vivo	lamb	Sarda × Comisana crossbreed, male	Chestnut, Tara, Mimosa *A. nigraa*, and Gambier	CT and HT	0–40	27,638	supplemented in diet	barley, alfalfa, wheat bran, molasses, and soybean meal
58	[65]	in situ	sheep	-	Quebracho	CT	0–50	21/28	added in diet	grass hay, grass cube, and whole barley
59	[66]	in situ	sheep	-	Quebracho	CT	0–50	21/28	added in diet	grass hay, grass cube, and whole barley
60	[67]	in situ	dairy cow	FH	Mimosa and Quebracho	CT	0–50	ns	added in silage	ryegrass, grass hay, grass silage, and concentrate
61	[68]	in situ	steer	Taleshi	Pistachio	HT	0–10	ns	added in diet (canola and soya bean meal)	alfalfa hay, wheat straw, barley grain, corn grain, wheat bran, and cottonseed meal
62	[69]	in vivo	lamb	Arabi, fat-tailed, male	Pomagranate-peel	CT	0–33.5	14/78	treated with recycle poultry bedding	alfalfa hay, wheat straw, corn silage, recycled poultry bedding, soybean meal, corn grain, barley, and wheat bran
63	[70]	in vivo	lamb	Swiss White Hill	Chestnut	HT	0–2	44,531	mixed in diet	hay and concentrate
64	[71]	in vivo	dairy cow	-	Tannin	HT	0–4.9	14/21	mixed in diet	grass–clover silage, meadow hay, and pelleted concentrate
65	[72]	in vivo	dairy cow	Polish FH	Lingonberry leaves	CT	0–4.83	21/26	added in diet	maize silage, lucerne silage, grass silage, beet pulp, brewer grain, rapeseed meal, and concentrate
66	[73]	in vivo	ewe	Assaf ewes	Tannin	Mixed and CT	0–10	14/28	supplemented in diet	alfalfa hay, concentrate
67	[74]	in vivo	ewe	Assaf ewes	Quebracho	CT	0–40	14/28	supplemented in diet	alfalfa hay, concentrate
68	[75]	in vivo	sheep and goat (boar)	Rambouillet and Spanish Boer, ewe	Quebracho	CT	0–100	15/18	mixed in diet	Sudan grass hay, corn, soybean meal, fish meal, and wheat straw
69	[76]	in vivo	sheep	Merino-Landschaf Crossbreed	Chestnut and Valonea	HT	0–20	ns/190	mixed in diet	ryegrass-based hay, barley grain, wheat grain, soybean meal, and molasses
70	[77]	in vivo and in situ	sheep and goat	-	Tannin	HT	0–110	14/24	sprayed to hay	grassland hay

**Table 2:** Descriptive statistics of the variables in the database were used to evaluate the influence of tannin extract supplementation on ruminant parameters.

Response Variables	Unit	n	Mean	SEM	Min	Max	Response Variables	Unit	n	Mean	SEM	Min	Max
Intake							IsoC_5_	mol/100 mol	81	2.16	5.82	0.09	53
DMI	kg/d	172	8.12	8.17	0.4	27.7	C_2_:C_3_		109	3.82	1.3	1.34	8.3
OMI	kg/d	80	6.8	7.46	0.3	24.6	Bacteria	log 10	12	6.77	0.26	6.47	7.2
CPI	kg/d	86	1.03	1.45	0	5.15	Protozoa	log 10	42	5.48	0.83	3.03	6.3
NDFI	kg/d	75	3.05	2.68	0.2	10	Feed disappearance						
DMI/BW^0.75^	g/kg	161	101	46.5	24	205	Ruminal protein	g/100 g	22	61.9	10.4	51.3	83
OMI/BW^0.75^	g/kg	83	85.5	39.6	27	188	Digested ruminal DM-N	g/100 g	22	61.7	16.9	15	85
CPI/BW^0.75^	g/kg	89	13.7	9.64	2.1	41.5	Digested ruminal OM-N	g/100 g	14	54.9	13.6	42	82
NDFI/BW^0.75^	g/kg	78	38.9	15.5	15	74.1	Duodenum protein	g/100 g	22	74.6	13.6	55.8	90
Digestibility							Intestinal protein	g/100 g	22	89.9	6.21	80	96
DMD	g/100 g	144	60.8	11.8	26	82.6	Blood plasma						
OMD	g/100 g	135	68.4	7.82	44	83.9	PUN	mg/dL	31	19	14	7.28	58
CPD	g/100 g	134	65.2	14.1	8	89.5	Albumin	g/dL	14	4.04	0.83	3.08	5.4
NDFD	g/100 g	165	50.3	17	0.2	79	N utilisation						
Performance							Milk N	g/100 g N	22	27.6	4.21	18.7	34
ADG	g/d	45	591	497	109	1920	Urine N	g/100 g N	85	47.7	46.9	16.4	459
ADG/DMI	g/kg	45	101	66.3	0.2	241	Faecal N	g/100 g N	83	39.1	13.2	20.9	83
GEI/BW^0.75^	kcal/kg	19	289	131	220	657	N retention	g/100 g N	67	21.6	10.3	0.6	39
DEI/BW^0.75^	kcal/kg	19	186	105	132	507	ENU	%	14	30.1	6.94	20	41
MEI/BW^0.75^	kcal/kg	40	56.4	61.2	0.1	142	Urinary purine						
Methane production							Allantoin	mmol/d	37	69	94.8	8.9	408
CH_4_	L	57	204	219	17	690	Uric acids	mmol/d	36	18.9	36.7	1	154
CH_4_/DMI	L/kg	51	24.7	7.87	6.7	42	Purine derivative	mmol/d	42	62.8	97.8	9.43	449
CH_4_/BW^0.75^	L/kg	57	2.53	1.23	0.6	5.51	Microbial N supply	g/d	47	34.6	29.9	3.41	91
Milk production and composition							EMPS	g/g	38	51.1	42.7	9.5	164
Milk	kg/d	65	21.7	13.1	0.7	40.8	DM kinetics degradability						
Milk/BW^0.75^	g/kg	63	0.19	0.08	0	0.31	A	%	59	28.2	14.9	7.67	75
Milk/DMI	kg/kg	69	1.34	0.47	0.3	2.98	B	%	59	62.9	20.1	12.6	90
FPCM	kg/d	16	26.9	17.4	0.9	56	*a* + *b*	%	67	90	16	31	102
Milk fat	g/100 g	63	4.42	1.23	2.9	7.69	C	/h	65	0.06	0.02	0.02	0.1
Milk protein	g/100 g	65	3.79	0.99	2.8	6.41	ERD 2%	kp_0.02_	24	72.2	7.76	53.4	82
Milk lactose	g/100 g	63	4.73	0.33	4	5.27	ERD 5%	kp_0.05_	44	56	14.4	27.2	91
Milk SNF	g/100 g	38	8.47	2.61	3.5	11.1	ERD 8%	kp_0.08_	24	47.6	10.7	27.2	63
Milk TSC	g/100 g	36	13.5	3.56	7.4	18.7	CP kinetics degradability						
Milk urea-N	mg/dL	35	21.5	9.73	9.4	44.7	*a*	%	73	23.4	16.3	1.2	75
Rumen fermentation							*b*	%	73	72.8	18.5	2.75	97
pH		123	6.54	0.32	5.8	7.43	*a* + *b*	%	73	96.1	13	57.6	120
NH_3_	mg/dL	109	18	8.4	3.2	39.4	c	/h	71	0.05	0.03	0.01	0.1
TVFA	mmol/L	107	94.4	29.1	40	158	ERD 2%	kp_0.02_	21	71	14.2	42.3	88
C_2_	mol/100 mol	109	65.5	7.8	47	79.8	ERD 5%	kp_0.05_	67	54.1	12.3	29.2	82
C_3_	mol/100 mol	109	19.1	5.33	9.5	36.8	ERD 8%	kp_0.08_	35	48.3	10.8	24.4	69
IsoC_4_	mol/100 mol	77	2.38	3.45	0.1	15.2	Ruminal N in situ degradability						
C_4_	mol/100 mol	109	11	4.41	1.2	26.2	ID	%	22	62.9	17.5	39	91
C_5_	mol/100 mol	86	1.24	0.81	0.2	3.82	RUP	%	13	40.6	11.4	24.4	60

DMI: dry matter intake; OMI: organic matter intake; CPI: crude protein intake; NDFI: neutral detergent fibre intake; BW^0.75^: metabolic body weight; DMD: digested dry matter; OMD: digested organic matter; CPD: digested crude protein; NDFD: digested neutral detergent fibre; ADG: average daily gain; GEI: gross energy intake; DEI: digestible energy intake; MEI: metabolizable energy intake; CH_4_: methane production; FPCM: fat protected corrected in milk; milk SNF: milk solid non-fat; milk TSC: milk total solid content; NH_3_: ammonia concentration; C_2_: acetate; C_3_: propionate; Iso-C_4_: isobutyrate; C_4_: butyrate; C_5_: valerate; Iso-C_5_: isovalerate; PUN: plasma urea-N concentration; ENU: efficiency of N utilisation; EMPS: efficiency of microbial protein synthesis; *a*: non-soluble degradable fraction; *b*: fractional degradation rate of the b fraction; *c*: degradation per -h [78]; *a* + *b*: potential degradation rate; ERD: effective rumen degradability; ID: in situ degradability; RUP: rumen undegradable protein; n: dietary treatments; SEM: standard error of means.

**Table 3:** Regression equations on the influence of tannin extract supplementation (T, in g/kg DM; independent factor) on ruminant intake, digestibility, ruminant performance, methane production, as well as milk production and milk composition.

Response Variables	Unit	n	Model	Parameter Estimates				Model Statistics			
				Intercept	SE Intercept	Slope	SE Slope	*p*-Value	RMSE	AIC	*p*-Value ct vs. ht
Intake											
DMI	kg/d	170	Q	8.95	1.05	−0.016	0.005	0.002	0.38	656	0.381
						0.0001	0.00006	0.021			
OMI	kg/d	79	L	7.17	1.40	−0.004	0.002	0.105	0.23	259	0.782
CPI	kg/d	84	L	1.11	0.27	−0.0004	0.0003	0.149	0.05	108	0.379
NDFI	kg/d	74	L	3.00	0.52	−0.003	0.001	0.025	0.14	142	0.280
DMI/BW^0.75^	g/kg	165	L	106	6.07	−0.09	0.02	<0.001	3.89	1256	0.123
OMI/BW^0.75^	g/kg	82	L	89.2	7.26	−0.051	0.03	0.058	2.90	618	0.797
CPI/BW^0.75^	g/kg	87	L	14.8	1.71	−0.013	0.004	0.005	0.84	432	0.203
NDFI/BW^0.75^	g/kg	77	L	39.5	2.92	−0.05	0.02	0.003	1.59	471	0.096
Digestibility											
DMD	g/100 g	116	Q	66.6	1.12	−0.14	0.03	<0.001	1.98	680	0.323
						0.0008	0.0003	0.003			
OMD	g/100 g	134	Q	70.3	1.10	−0.13	0.02	<0.001	1.96	788	0.568
						0.0007	0.0003	0.006			
CPD	g/100 g	124	Q	68.4	2.09	−0.24	0.03	<0.001	2.25	794	0.337
						0.002	0.0005	0.001			
NDFD	g/100 g	137	Q	57.5	1.64	−0.15	0.03	<0.001	2.59	890	0.044
						0.0009	0.0003	0.009			
Performance											
ADG	g/d	45	L	558	145	−0.48	0.84	0.575	70.4	579	0.135
ADG/DMI	g/kg	45	Q	99.5	19.2	0.96	0.55	0.092	19.4	458	0.376
						−0.03	0.01	0.007			
GEI/BW^0.75^	kcal/kg	19	L	296	56.9	−0.45	0.68	0.525	26.5	202	N.a.
DEI/BW^0.75^	kcal/kg	19	L	200	44.5	−0.85	0.73	0.264	28.3	201	N.a.
MEI/BW^0.75^	kcal/kg	40	L	53.9	18.6	0.0008	0.008	0.919	1.21	224	N.a.
Methane production											
CH_4_	L	57	L	217	49.3	−0.51	0.39	0.200	30.9	656	0.047
CH_4_/DMI	L/kg	51	L	26.4	1.94	−0.10	0.02	<0.001	1.87	292	0.051
CH_4_/BW^0.75^	L/kg	57	L	2.74	0.27	−0.009	0.003	0.007	0.25	111	0.046
Milk production and composition											
Milk yield	kg/d	65	Q	21.7	2.61	−0.04	0.02	0.081	1.02	385	0.999
						0.0003	0.0002	0.083			
Milk yield/BW^0.75^	g/kg	63	L	186	17.0	0.02	0.09	0.859	0.29	−234	0.809
Milk yield/DMI	g/kg	69	L	1337	90.3	0.56	0.65	0.399	1.67	31.1	0.611
FPCM	kg/d	16	Q	24.9	6.37	−0.09	0.02	0.002	0.31	95.7	0.300
						0.002	0.0003	<0.001			
Milk fat	g/100 g	63	L	4.55	0.25	−0.0003	0.001	0.776	0.12	83.2	0.664
Milk protein	g/100 g	65	L	3.84	0.20	−0.0003	0.001	0.582	0.06	21.1	0.094
Milk lactose	g/100 g	63	L	4.74	0.07	−0.00005	0.0004	0.904	0.04	−60.9	0.022
Milk SNF	g/100 g	38	L	8.75	0.72	−0.009	0.002	<0.001	0.14	78.2	0.650
Milk TSC	g/100 g	36	L	14.1	1.01	−0.009	0.003	0.006	0.20	97.2	0.728
Milk urea-N	mg/dL	35	L	22.8	2.64	−0.047	0.013	0.001	0.85	188	0.339

DMI: dry matter intake; OMI: organic matter intake; CPI: crude protein intake; NDFI: neutral detergent fibre intake; BW^0.75^: metabolic body weight; DMD: dry matter digestibility; OMD: organic matter digestibility; CPD: crude protein digestibility; NDFD: neutral detergent fibre digestibility; ADG: average daily gain; GEI: gross energy intake; DEI: digestible energy intake; MEI: metabolizable energy intake; CH_4_: methane production; FPCM; fat protected corrected in milk; milk SNF: milk solid non-fat; milk TSC; milk total solid content; L: linear; Q: quadratic; SE; standard of errors; RMSE: root mean square of errors; AIC: Akaike information criterion; CT: condensed tannin effect; HT; hydrolysable tannin effect.

**Table 4:** Regression equations on the influence of tannin extract supplementation (T, in g/kg DM; independent factor) on rumen fermentation profile and feed disappearance in the rumen.

Response Variables	Unit	n	Model	Parameter Estimates				Model Statistics			
				Intercept	SE Intercept	Slope	SE Slope	*p*-Value	RMSE	AIC	*p*-Value ct vs. ht
Rumen fermentation profile											
pH		122	L	6.50	0.05	0.0003	0.0004	0.502	0.10	−33.5	0.104
NH_3_	mg/dL	108	L	19.4	1.40	−0.08	0.01	<0.001	1.88	625	0.155
VFA	mmol/L	106	L	98.9	4.81	−0.04	0.03	0.162	6.84	875	0.628
C_2_	mol/100mol	108	L	65.3	1.30	−0.020	0.006	<0.001	1.36	573	0.016
C_3_	mol/100 mol	108	L	19.0	0.88	0.017	0.005	<0.001	1.15	522	0.287
Iso-C_4_	mol/100 mol	77	Q	2.42	0.72	0.02	0.01	0.008	0.42	262	0.755
						−0.0002	0.0001	0.002			
C_4_	mol/100 mol	108	L	11.3	0.72	0.001	0.006	0.815	1.38	533	0.010
C_5_	mol/100 mol	85	Q	1.22	0.16	−0.008	0.002	0.001	0.15	98.9	0.625
						0.0001	0.00002	<0.001			
Iso-C_5_	mol/100 mol	81	L	2.47	0.94	−0.0028	0.016	0.862	4.05	522	0.604
C_2_:C_3_		108	L	3.83	0.21	−0.006	0.001	<0.001	0.27	214	0.202
Bacteria	log 10	12	L	6.71	0.11	0.00024	0.0005	0.663	0.06	9.20	N.a.
Protozoa	log 10	42	L	5.31	0.22	−0.0012	0.0006	0.058	0.10	30.7	0.714
Feed disappearance											
Ruminal protein	g/100 g	22	Q	69.5	3.17	−0.72	0.26	0.015	2.17	140	N.a.
						0.01	0.01	0.022			
Digested ruminal DM-N	g/100 g	22	L	72.2	4.72	−0.43	0.08	<0.001	5.63	165	N.a.
Digested ruminal OM-N	g/100 g	14	L	62.1	4.98	−0.31	0.07	0.002	3.61	98.2	N.a.
Duodenum protein	g/100 g	22	L	76.0	6.36	−0.03	0.04	0.480	2.31	134	N.a.
Intestinal protein	g/100 g	22	L	91.9	2.88	−0.03	0.01	0.038	0.73	91.3	N.a.

NH_3_: ammonia concentration; C_2_: acetate; C_3_: propionate; Iso-C_4_: isobutyrate; C_4_: butyrate; C_5_: valerate; Iso-C_5_: isovalerate; L: linear; Q: quadratic; SE; standard of errors; RMSE: root mean square of errors; AIC: Akaike information criterion; CT: condensed tannin effect; HT; hydrolysable tannin effect.

**Table 5:** Regression equations on the influence of tannin extract supplementation (T, in g/kg DM; independent factor) on ruminant blood plasma, percentage of N utilisation, and ruminant urinary purine.

Response Variables	Unit	n	Model	Parameter Estimates				Model Statistics			
				Intercept	SE Intercept	Slope	SE Slope	*p*-Value	RMSE	AIC	*p*-Value ct vs. ht
Blood plasma											
PUN	mg/dL	31	Q	20.7	3.33	−0.19	0.05	0.002	0.98	177	0.089
						0.005	0.001	0.002			
Albumin	g/dL	14	L	4.01	0.48	−0.0013	0.003	0.698	0.14	20.7	0.060
N utilisation											
Milk N	g/100 g N	22	L	26.9	1.46	−0.0023	0.02	0.910	0.73	102	0.742
Urine N	g/100 g N	85	L	44.6	7.82	0.26	0.24	0.286	31.4	891	0.891
Faecal N	g/100 g N	83	L	35.0	2.53	0.18	0.02	<0.0001	2.47	535	0.802
N retention	g/100 g N	67	Q	21.7	2.13	0.23	0.06	<0.001	2.23	421	0.012
						−0.004	0.001	<0.001			
ENU	%	14	Q	30.0	3.32	0.42	0.20	0.079	3.09	99.9	N.a.
						−0.008	0.004	0.070			
Urinary purine											
Allantoin	mmol/d	37	L	76.0	29.6	−0.40	0.23	0.105	17.94	374	N.a.
Uric acids	mmol/d	36	L	27.1	11.3	−0.09	0.05	0.084	3.61	273	<0.001
Purine derivative	mmol/d	42	L	65.6	29.2	−0.40	0.23	0.097	18.83	427	0.869
Microbial N supply	g/d	47	L	36.9	7.71	0.02	0.03	0.539	2.48	332	0.676
EMPS	g/g	38	L	58.3	11.7	−0.56	0.26	0.043	18.27	370	<0.001

PUN: plasma urea-N concentration; ENU: efficiency of N utilisation; EMPS: effectiveness of microbial protein synthesis; L: linear; Q: quadratic; SE: standard of errors; RMSE: root mean square of errors; AIC: Akaike information criterion; CT: condensed tannin effect; HT: hydrolysable tannin effect.

**Table 6:** Regression equations on the influence of tannin extract supplementation (T, in g/kg DM; independent factor) on in situ dry matter kinetic degradability and protein kinetic degradability of ruminants.

Response Variables	Unit	n	Model	Parameter Estimates				Model Statistics			
				Intercept	SE Intercept	Slope	SE Slope	*p*-Value	RMSE	AIC	*p*-Value ct vs. ht
DM kinetics degradability											
*a*	%	59	Q	30.7	3.73	−0.14	0.03	<0.001	2.85	402	0.006
						0.0005	0.0002	0.020			
*b*	%	59	Q	56.7	5.08	0.14	0.03	0.001	2.95	418	0.072
						−0.0008	0.0002	0.001			
*a* + *b*	%	67	L	86.9	3.58	−0.04	0.009	<0.001	1.96	421	0.421
*c*	%/h	65	L	0.06	0.005	−0.0002	0.00004	<0.001	0.01	−324	0.941
ERD 2%	kp _0.02_	24	L	79.0	1.85	−0.36	0.03	<0.001	2.19	133	0.025
ERD 5%	kp _0.05_	44	Q	61.7	4.04	−0.33	0.05	<0.001	3.45	311	0.160
						0.001	0.0003	<0.001			
ERD 8%	kp _0.08_	24	L	55.6	2.81	−0.42	0.03	<0.001	2.23	138	0.006
CP kinetics degradability											
*a*	%	73	Q	30.9	3.80	−0.30	0.03	<0.001	2.79	485	<0.001
						0.001	0.0002	<0.001			
*b*	%	73	Q	64.0	4.42	0.28	0.06	<0.001	5.10	555	<0.001
						−0.002	0.0003	<0.001			
*a* + *b*	%	73	L	95.6	3.04	−0.09	0.02	<0.001	3.70	486	0.003
*c*	%/h	71	Q	0.06	0.01	−0.0006	0.0001	<0.001	0.01	−320	0.822
						0.000002	0.00	0.001			
ERD 2%	kp _0.02_	21	Q	81.1	6.69	−0.72	0.09	<0.001	1.64	128	N.a.
						0.007	0.002	0.001			
ERD 5%	kp _0.05_	67	Q	60.8	2.72	−0.37	0.04	<0.001	3.37	456	0.281
						0.0011	0.0002	<0.001			
ERD 8%	kp _0.08_	35	Q	58.1	3.91	−0.83	0.07	<0.001	1.49	188	N.a.
						0.007	0.001	<0.001			
Ruminal N in situ degradability											
ID	%	22	L	65.4	8.79	−0.08	0.02	0.001	3.86	152	0.306
RUP	%	13	L	32.3	5.13	0.21	0.02	<0.001	2.10	80.3	N.a.

*a*: non-soluble degradable fraction; *b*: fractional degradation rate of the b fraction; *c*: degradation per -h (Ørskov and McDonald, 1979); *a* + *b*: potential degradation percentage; ERD: effective rumen degradability; ID: in situ degradability; RUP: rumen undegradable protein; L: linear; Q: quadratic; SE: standard of errors; RMSE: root mean square of errors; AIC: Akaike information criterion; CT: condensed tannin effect; HT: hydrolysable tannin effect.

## Data Availability

Not available.
